# Activation of the muscle‐to‐brain‐axis reduces amyloid plaque accumulation and rescues behavioral deficits by enhancing synaptic integrity and neurotrophic signaling in a mouse model of Alzheimer’s disease

**DOI:** 10.1002/alz.094857

**Published:** 2025-01-09

**Authors:** Hash Brown Taha, Allison Birnbaum, Ian Matthews, Karel Aceituno, Jocelyne Leon, Max A Thorwald, Jose A Godoy‐Lugo, Constanza J Cortes

**Affiliations:** ^1^ USC Leonard Davis School of Gerontology, Los Angeles, CA USA

## Abstract

**Background:**

Alzheimer’s disease (AD) is associated with complex pathophysiology including synaptic dysregulation, compromised neurotrophic signaling, deficits in autophagic flux and neuroinflammation). Skeletal muscle regulates many brain functions relevant to aging, by activating the muscle‐to‐brain axis through the secretion of skeletal muscle originating factors (myokines) with cellular‐modifying, neuro and geroprotective properties. Our group developed transgenic mice that overexpress the skeletal muscle human Transcription Factor EB (TFEB), a master regulator of lysosomal‐to‐nucleus signaling, resulting in enhanced proteostasis and neuroprotection in a Tau mouse model. However, the precise mechanisms remain unknown. Therefore, we further validated these effects in an AD amyloid β (Aβ) mouse model and investigated the underlying mechanism.

**Method:**

We crossbred female 5xFAD mice, carrying 5 AD familial mutations, with transgenic mice that overexpress human TFEB to create 5xFAD;cTFEB;HSA‐Cre (3FA) mice. At 4 and 8 months of age, we analyzed Aβ plaque accumulation through immunohistochemistry and conducted western blot analysis for multiple synaptic markers, growth factors, autophagic/lysosomal regulators, and myokines across the muscle‐to‐brain axis. We also performed a battery of neurocognitive tests (open field, the Barnes maze, and fear conditioning) at 8 months of age, when this model has previously been reported to demonstrate robust cognitive impairment.

**Result:**

Skeletal muscle‐targeted TFEB expression reduced Aβ plaque accumulation in cortices of 4‐month‐old female mice. Furthermore, muscle‐TFEB expression altered synaptic‐associated gene transcriptional signatures in hippocampi, and rescued behavioral deficits in 8‐month‐old female 5xFAD mice. Western blots of cortex from 8‐month‐old female 3FA mice confirmed a rescue of several synaptic markers including SNAP25, synaptophysin I, synaptotagmin I and PSD95, neurotrophic factors such as BDNF and autophagic/lysosomal regulators such as Cathepsin D and B, prosaposin (PSAP) and saposin C. Levels of PSAP (a recently identified exercise‐responsive myokine) were also increased in skeletal muscle, plasma and cortices, suggesting that PSAP may act as a novel myokine involved in muscle‐to‐brain rescue mechanisms. scle, plasma and cortices of 8‐month‐old female mice, suggesting that PSAP may act as a novel myokine involved in muscle‐to‐brain rescue mechanisms.

**Conclusion:**

Skeletal muscle directly regulates CNS function and health in the 5xFAD model regulating synaptic integrity, neurotrophic signaling and autophagic flux, potentially through release of CNS‐targeting myokines.

